# *Quadrella incana* (Capparaceae) Leaf Extract Enhances Proliferation and Maintenance of Neural Stem/Progenitor Cells through Upregulating Glycolytic Flux and Redox Potential

**DOI:** 10.1155/2020/5963037

**Published:** 2020-04-25

**Authors:** Mingyu Kang, Jin-Hwa Cho, Tae-Jun Kim, Sang Min Lee, Hyeon Ji Kim, Indiana Coronado, Dong-Keun Yi, Do-Yeon Kim

**Affiliations:** ^1^Department of Pharmacology, School of Dentistry, Kyungpook National University, Daegu 41940, Republic of Korea; ^2^Herbarium (HULE), National University Autonomous of Nicaragua-Leon, Leon 68, Nicaragua; ^3^International Biological Material Research Center, Korea Research Institute of Bioscience and Biotechnology, Daejeon 34141, Republic of Korea; ^4^Department of Pharmacology, School of Dentistry, Brain Science and Engineering Institute, Kyungpook National University, Daegu 41940, Republic of Korea

## Abstract

Neural stem/progenitor cells (NSPCs) are self-renewing, multipotent cells located in the embryonic and adult central nervous system (CNS). Extensive preclinical and clinical studies have shed light on the potential of stem cell replacement therapy for various neurodegenerative diseases. The key prerequisite for the success of these clinical applications is the procurement of a sufficient number of high-quality NSPCs. In this study, we explored the biological activity of *Quadrella incana* leaf in NSPC homeostasis. We showed that the leaf extract of *Quadrella incana* upregulated NSPC marker and proliferative potential. On the other hand, *Quadrella incana* leaf suppressed spontaneous unintended NSPC differentiation. Mechanistically, *Quadrella incana* leaf contributed to the maintenance of NSPCs by upregulating glycolytic flux and redox potential.

## 1. Introduction

Neural stem/progenitor cells (NSPCs) are undifferentiated, multipotent cells originating in the embryonic and adult central nervous system (CNS). During embryonic development, NSPCs have the capacity to expand, self-renew, and produce neurons and glial cells of mammalian CNS [1]. Although the research on adult neurogenesis remains controversial [2], it has been known that adult neural stem cells mainly residing within the subgranular zone of the hippocampal dentate gyrus and the subventricular zone of the lateral ventricle wall actively produce functional neurons throughout life [3]. Extensive preclinical trials over the past few decades have suggested the potential of stem cell replacement therapy for various neurological disorders [4, 5]. Furthermore, recent clinical trials have been partially reported on neural stem cell transplantation for the treatment of ALS [6] and chronic spinal cord injury [7]. The most essential prerequisite for the success of these clinical applications is the procurement of a sufficient number of high-quality neural stem cells.

Several mechanisms have been suggested to underlie the homeostatic regulation of NSPCs. Among these, metabolic control of NSPCs has been extensively studied. Compared to differentiated neurons, NSPCs are highly reliant on the anaerobic glycolytic pathway for energy production and survival. Under normoxic conditions, LDH activity and lactate production were found to be enhanced in NSPCs compared to neurons [8]. During neuronal differentiation from NSPCs, the expression of most glycolysis genes, particularly glucose transporters encoded by Glut1/3 as well as Ldha encoding lactate dehydrogenase A, was reduced. Consistent with this result, the levels of glycolysis intermediates were dramatically lower in neurons compared to NSPCs. In contrast, the metabolic regulators PGC-1*α* and ERR*γ* were significantly increased during neuronal differentiation to maintain the expression of TCA and mitochondrial respiratory complex genes [9]. The metabolic conversion from aerobic glycolysis to mitochondrial OXPHOS is essential for neuronal differentiation. FoxO3-null NSPCs displayed a reduction in mitochondria-localized HK2 via downregulated AKT signaling, leading to a diminished proliferative potential [10]. PINK1-deficient NSPCs showed mitochondrial defects and elevated glycolysis, and the differentiation/maturation of newborn neurons in PINK1^−/−^ mice was clearly compromised [11]. Constitutive expression of the core glycolytic genes, HK2 and LDHA, was found to induce neuronal cell death, suggesting that blockade of glycolytic flux is critical for proper neuronal differentiation and survival [9].

Another important feature governing NSPC maintenance is resistance towards oxidative stress. Previous studies have reported that NSPC cultures spontaneously produced superoxide anion radicals, which suppressed the proliferation of NSPCs. Consistently, mice lacking Sod2 that scavenges oxygen radicals showed significantly fewer proliferative NSPCs *in vivo* [12]. Another study demonstrated that NSPCs have a capacity to maintain reactive oxygen species (ROS) at low levels, and ROS deposition accompanies the differentiation of NSPCs into neurons [13]. While signaling molecules that promoted NSPC self-renewal also caused cells to exist in a reduced state, extrinsic factors that induced NSPC differentiation led to a more oxidized intracellular state, suggesting that redox state could be a critical modulator of the cell fate decision between self-renewal and differentiation [14]. In line with these findings, oxidative stress was shown to trigger the progressive loss of the stemness in NSPCs and promote spontaneous neuronal differentiation [15].

Historically, human has used natural products for medical purposes [16]. The anticancer drug Taxol is the most prominent example of a drug derived from the plant. However, given the possibility of positive interactions between components in crude extracts or among different medicinal plants, the crude extract(s) of one or more specific plant(s) could be used in certain situations [17]. In this study, we explored the biological activity of the *Quadrella incana* leaf. We showed that the extract of *Quadrella incana* leaf upregulated the NSPC marker and increased the cellular proliferative potential. Simultaneously, *Quadrella incana* leaf suppressed spontaneous NSPC differentiation. Mechanistically, *Quadrella incana* leaf contributed to the maintenance of NSPCs by upregulating glycolytic flux and redox potential.

## 2. Materials and Methods

### 2.1. Neural Stem Cell Preparation and Differentiation

Primary NSPCs were isolated from the brain of day 10.5 mouse embryos and maintained in N2 culture medium supplemented with 20 ng/ml EGF and bFGF. For differentiation induction, NSPCs were dissociated into single cells using TrypLE (Life Technologies) and plated on polyornithine and fibronectin-coated plates in an N2 culture medium including 1% fetal bovine serum (FBS) and B27 Supplements (Life Technologies) without growth factors for 3 days.

### 2.2. Preparation of *Quadrella incana* (Kunth) Iltis & Cornejo (Capparaceae) Leaf Extract

*Quadrella incana* (Kunth) Iltis & Cornejo was collected in Ocotal city, Nueva Segovia province, in Nicaragua, and identified in 2014 by Indiana Coronado of the Herbarium, National Autonomous University of Nicaragua at Leon. A voucher specimen (accession number KRIB 0058006) of the retained material is preserved at the herbarium of KRIBB. The dried and refined whole plant of *Quadrella incana* (53 g) was extracted with 1 l of 99.9% (*v*/*v*) methanol with repeated sonication (15 min) and resting (2 h) for 3 days at 45°C. The resultant product was filtered with nonfluorescent cotton and concentrated by rotary evaporator (N-1000SWD, EYELA) under reduced pressure at 45°C. A final total of 8.55 g methanol extract of *Quadrella incana* was obtained upon freeze-drying. Dried *Quadrella incana* leaf was dissolved in DMSO at a stock concentration of 100 mg/ml.

### 2.3. Protein Preparation and Immunoblot Analysis

Cells were disrupted directly with laemmli buffer (60 mM Tris-HCl (pH 6.8), 2% (*w*/*v*) SDS, 10% (*v*/*v*) glycerol, 0.02% (*w*/*v*) bromophenol blue), followed by sonication and heat denaturation at 95°C. Samples were fractionated by SDS-PAGE and transferred to a PVDF membrane. After blocking membranes with 5% nonfat dried milk in TBST (10 mM Tris, pH 8.0, 150 mM NaCl, 0.5% Tween 20) for 30 min, the membrane was washed with TBST and incubated with antibodies against Bmi1 (1 : 1,000, Bethyl Laboratories), *β*III tubulin (1 : 1,000, Abcam), phospho-Histone 3 at Ser 10 (1 : 1,000, Cell Signaling), *β* Actin (1 : 5,000, Sigma Aldrich), GAPDH (1 : 1,000, Cusabio), Fubp1 (1 : 1,000, Abcam), Sox2 (1 : 1,000, Cusabio), Prdx3 (1 : 1,000, Bethyl Laboratories), phospho-mTOR (1 : 1,000, Cell Signaling), total mTOR (1 : 1,000, Cell Signaling), FoxO1 (1 : 1,000, Thermo Fisher Scientific), FOXO3 (1 : 1,000, Thermo Fisher Scientific), phospho AKT (1 : 1,000, Thermo Fisher Scientific), and total AKT (1 : 1,000, Cell Signaling) overnight at 4°C. The next day, membranes were washed three times (10 min each) with TBST and incubated with horseradish peroxidase-conjugated antimouse (1 : 10,000, Bethyl Laboratories) or antirabbit antibodies (1 : 5,000, Bethyl Laboratories) for 1 hour. Membranes were washed with TBST, and signals were detected with D-Plus^TM^ ECL Femto system (Dongin LS). Quantification of Western blots was performed with ImageJ.

### 2.4. Immunofluorescence

Cells were fixed with 4% paraformaldehyde and permeabilized with 0.2% Triton X-100/PBS for 15 min each at room temperature. After blocking samples with 2% BSA/PBS for 30 min, cells were subjected to immunofluorescence staining with anti-Nestin (1 : 100, Novus Biologicals), anti-FoxO1 (1 : 200, Thermo Fisher Scientific), and anti-*β*III tubulin (1 : 200, Abcam) primary antibodies overnight at 4°C. The next day, cells were washed with PBS and incubated with Flamma®552- or Flamma®488-conjugated goat antirabbit IgG (Bioacts) or goat antimouse IgG (Bioacts) for 30 min at room temperature. Fluorescence signals were visualized with the EVOS FL Auto Imaging System (Thermo Fisher Scientific).

### 2.5. Quantitative Real-Time RT-PCR

Total RNA was isolated by using an RNA extraction kit (Favorgen, Taiwan). 250 ng of total RNA was treated with RNase-free DNase (Sigma-Aldrich, St. Louis, MO, USA) for 15 min. After the inactivation of DNase with EDTA and heating, RNA was reverse transcribed using First Strand cDNA Synthesis Kit (Thermo Fisher Scientific) according to the manufacturer's instructions. Quantitative RT-PCR was performed on cDNA samples using the Luna qPCR master mix (NEB, Ipswich, MA, USA) by using the Mic qPCR Cycler (Bio Molecular Systems, Australia). Relative mRNA levels are presented as values of 2^[Ct(Rpl32)–Ct(gene of interest)]. For data presentation, the mRNA level in the control cell was set to 1. The sequences of the forward and reverse primers are shown in Table 1.

### 2.6. Transfection and Reporter Assays

For Notch reporter plasmid transfection, NSPCs were dissociated and plated in an N2 culture medium including EGF and bFGF. After a 24-hour incubation, firefly luciferase mock vector or 4X CSL-luciferase reporter plasmid (a gift from Raphael Kopan, Addgene plasmid # 41726) [18] was cotransfected with a TK-renilla plasmid using Lipofectamine 3000 (Life Technologies) according to the manufacturer's instruction. At 12 hours posttransfection, cells were treated with vehicle or 100 *μ*g/ml *Quadrella incana* leaf extract for an additional 24 hours. Harvested cells were subjected to the luciferase activity measurement. Relative luciferase activity was determined as the ratio of firefly to Renilla activity. For data presentation, the luciferase activity of the pGL3 mock-negative control vector was set to 1.

### 2.7. GSH/GSSG Measurement

Primary NSPCs were maintained in N2 culture media supplemented with 20 ng/ml EGF and bFGF. At 3 days after treatment with vehicle or 100 *μ*g/ml *Quadrella incana* leaf extract, growth media was removed and cells were washed with PBS. Total glutathione and GSSG were assayed in triplicate with GSH/GSSG-Glo™ kit (Promega, Madison, WI, USA), following the manufacturer's instructions. The GSH/GSSG ratio was calculated as follows: [luminescence of total glutathione–luminescence of GSSG]/[luminescence of GSSG/2].

### 2.8. Lactate Level Measurement

To determine the intracellular lactate levels, cell lysates were prepared by washing with PBS, lysing with 0.6 N HCl, and neutralizing with 1 M Tris base. To analyze the lactate levels in culture media, the medium was diluted 500-fold in PBS. The intra- or extracellular lactate level was determined using Lactate-Glo™ kit (Promega, Madison, WI, USA), according to the manufacturer's instructions.

### 2.9. Statistical Analysis

The unpaired two-tailed Student's *t* test was used for experiments comparing two datasets unless otherwise noted. All results are expressed as mean ± s.e.m. GraphPad Prism software (version 6, San Diego, CA) was used for all statistical analyses. Differences were considered significant when ∗*P* < 0.05, ∗∗*P* < 0.01, and ∗∗∗*P* < 0.001.

## 3. Results

### 3.1. *Quadrella incana* Leaf Extract Enhanced the Proliferation and Stemness of NSPCs

**Figure 1:**
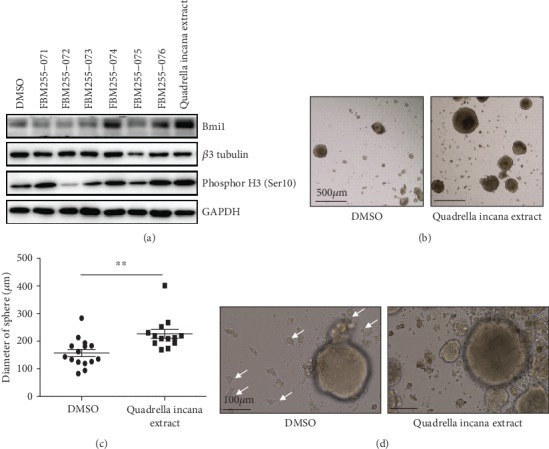
*Quadrella incana* leaf extract enhanced the proliferation and inhibited spontaneous differentiation of NSPCs in proliferating condition. (a) Western blot analysis of Bmi1, *β*III tubulin, and phospho-Histone 3 (Ser 10) in NSPCs kept under proliferating media upon treatment for 3 days with DMSO, *Quadrella incana* leaf extract, or six different plant crude extracts. GAPDH was used as a loading control. GAPDH was used as a loading control. (b) Sphere-forming capacity of NSPCs treated with vehicle (DMSO) or 100 *μ*g/ml *Quadrella incana* leaf extract for 3 days. Scale bar = 500 *μ*m. (c) Average diameters of suspended spheres treated with DMSO or 100 *μ*g/ml *Quadrella incana* leaf extract for 3 days. The unpaired two-tailed Student's *t* test was used to determine the statistical significance. ∗∗*P* < 0.01. (d) Morphology of NSPCs kept under proliferating media after treatment of DMSO or 100 *μ*g/ml *Quadrella incana* leaf extract for 3 days. Arrows indicate cells attached to the bottom of the plate.

To discover potential bioactivities related to NSPC maintenance, we first performed phytochemical screening with crude extracts from seven plant species. To focus on the proliferation and stemness of NSPCs in this study, we used NSPCs derived from early-stage (E10.5) mouse embryo, which retains a high proliferative capacity. Although NSPCs are multipotent cells that differentiate into neurons, astrocytes, and oligodendrocytes, E10.5 NSPCs give rise to neurons, not glial cells, after short-term culturing. Primary NSPCs were incubated with seven different extracts at the concentration of 100 *μ*g/ml for 3 days under a proliferating condition. Interestingly, *Quadrella incana* leaf extract significantly upregulated the protein level of Bmi1, which promotes NSPC self-renewal and proliferation [19, 20]. Consistently, we found the induction of the mitotic marker, phospho-Histone 3 at Ser 10 upon *Quadrella incana* leaf extract treatment. In contrast, differentiated neuronal marker *β*III tubulin was downregulated upon treatment with this extract, suggesting that *Quadrella incana* leaf has a suppressive effect on spontaneous differentiation of NSPCs (Figure 1(a)). Morphologically, the average sphere size of cultured NSPCs was much larger in *Quadrella incana* leaf extract-treated cells than vehicle-treated cells (Figures 1(b) and 1(c)). Notably, we observed some percentage of cells attached to the bottom of the plate when they were kept in proliferating media including DMSO for 3 days. However, the addition of *Quadrella incana* leaf extract inhibited this attachment, suggesting that it may contribute to the maintenance of NSPC stemness (Figure 1(d)). Facilitation of NSPC proliferation by *Quadrella incana* leaf extract was additionally confirmed by CCK-8 assay (Supplementary Fig 1A) and immunostaining of phospho-Histone 3 at Ser10 (Supplementary Fig 1B and 1C). Together, our data suggests that *Quadrella incana* leaf extract upregulated the proliferation and stemness of NSPCs.

### 3.2. *Quadrella incana* Leaf Extract Did Not Alter the Differentiation of NSPCs

**Figure 2:**
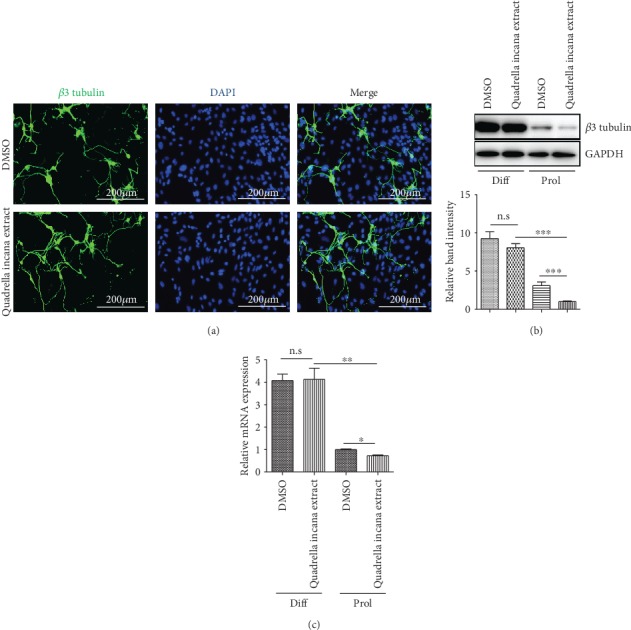
*Quadrella incana* leaf extract did not suppress the differentiation of NSPCs in the differentiating condition. (a) Immunofluorescence analysis of *β*III-tubulin (green) expression in DMSO- or *Quadrella incana* leaf extract-treated and differentiated NSPCs. Nuclear DAPI (4′,6-diamidino-2-phenylindole) staining is in blue. Scale bars = 200 *μ*m. (b) Western blot analysis of *β*III tubulin in NSPCs upon vehicle or *Quadrella incana* leaf extract treatment under proliferating condition (prol) or differentiating condition (diff). The relative band intensity of *β*III tubulin protein is shown on the graph below. (c) mRNA expression of Tubb3 in NSPCs upon vehicle or 100 *μ*g/ml *Quadrella incana* leaf extract treatment under proliferating condition (prol) or differentiating condition (diff) was determined by quantitative real-time PCR (qRT-PCR). Values are means ± s.e.m. ∗*P* < 0.05, ∗∗*P* < 0.01, ∗∗∗*P* < 0.001.

Given the stemness-maintaining function of *Quadrella incana* leaf extract under a proliferating condition, we next tested whether this plant extract could fully suppress the differentiation of NSPCs. To this end, primary NSPCs were differentiated for 3 days in the presence of DMSO or *Quadrella incana* leaf extract. Immunostaining for the neuron-specific marker *β*III tubulin demonstrated that the morphology and density of neuronal cells were not altered by *Quadrella incana* leaf extract treatment (Figure 2(a)). Consistent with this data, *Quadrella incana* leaf extract did not change the level of *β*III tubulin protein accumulation under the differentiation condition (Figure 2(b)). As shown in Figure 1(a), *Quadrella incana* leaf extract lowered *β*III tubulin protein level under the proliferating condition, confirming that it had an inhibitory effect on spontaneous differentiation of NSPCs. Consistent with the immunoblotting result, *Quadrella incana* leaf extract downregulated Tubb3 mRNA expression in the proliferating condition but showed a negligible effect on Tubb3 transcripts in the differentiation condition (Figure 2(c)). Together, our data suggests that *Quadrella incana* leaf could suppress unintended differentiation of NSPCs but could not fully block the induced differentiation of NSPCs.

### 3.3. Glycolytic Flux and Redox Homeostasis Are Important for the Proliferation of NSPCs

**Figure 3:**
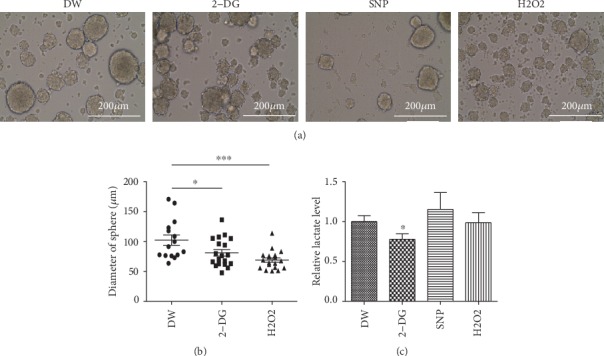
Disruption of glycolytic flux or upregulation of oxidative stress suppresses NSPC proliferation. (a) Sphere-forming capacity of NSPCs treated with vehicle (DW) or the indicated drugs for 24 hrs. 2-DG, 2-Deoxy-D-glucose (5 mM); SNP, sodium nitroprusside (200 *μ*M), H2O2, hydrogen peroxide (200 *μ*M). Scale bar = 200 *μ*m. (b) Average diameters of suspended spheres treated with DW or the indicated drugs for 24 hrs. The unpaired two-tailed Student's *t* test was used to determine the statistical difference. ∗*P* < 0.05, ∗∗∗*P* < 0.001. (c) Relative lactate levels in medium of proliferating NSPCs after treatment with DW or the indicated drugs for 24 hrs. The relative lactate level of DW-treated cells was set to 1. ∗*P* < 0.05.

Increasing evidence suggests that oxidative glycolysis and redox balance are critical mechanisms contributing to the proliferation and stemness of NSPCs. To confirm this, we treated NSPCs with the glycolytic inhibitor 2-deoxy-D-glucose (2-DG) and the oxidative stress inducers sodium nitroprusside (SNP) and hydrogen peroxide. Indeed, the average sphere size of cultured NSPCs was significantly reduced when glycolysis was inhibited or oxidative stress was induced (Figures 3(a) and 3(b)). Notably, when we challenged proliferating NSPCs with SNP, most cells tended to adhere to the bottom of the plate, suggesting that excessive nitric oxide donated by SNP caused cells to lose their sphere-forming capability. Although 2-DG treatment lowered lactate levels as expected, neither SNP nor hydrogen peroxide reduced lactate production, indicating that oxidative stress inducers decreased the proliferative potential of NSPCs independent of glycolytic inhibition (Figure 3(c)).

### 3.4. *Quadrella incana* Leaf Extract Contributed to the Redox Homeostasis of NSPCs

Given the importance of glycolytic flux and redox balance for the homeostasis of NSPCs, we explored the critical factors contributing to the proliferation and stemness maintenance of NSPCs. A previous study reported that FoxO family members cooperatively modulate NSPC proliferation and self-renewal [21]. Among FoxO genes, FoxO1 and FoxO3 are highly expressed in undifferentiated NSPCs, and they seem to favor NSPC proliferation over neuronal differentiation [10, 22]. Furthermore, these two genes are well-known critical factors in redox regulation [23].

**Figure 4:**
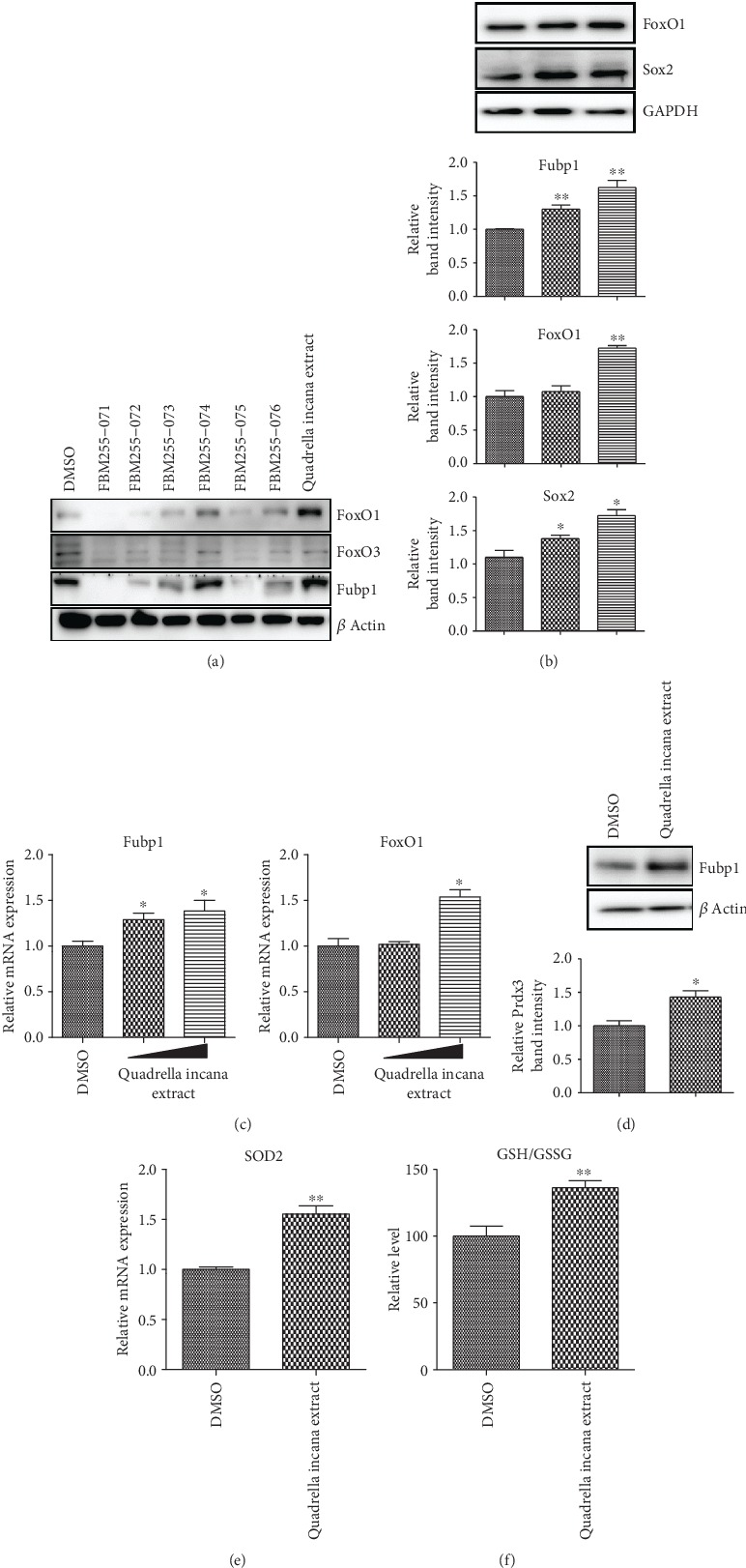
*Quadrella incana* leaf extract improved the oxidation-reduction potential of NSPCs by upregulating ROS scavenging genes. (a) Western blot analysis of FoxO1, FoxO3, and Fubp1 in NSPCs kept under proliferating media after treatment for 3 days with DMSO, *Quadrella incana* leaf extract, or six different plant crude extracts. *β* actin was used as a loading control. (b) Western blot analysis of Fubp1, FoxO1, and Sox2 in proliferating NSPCs after treatment with DMSO, 20 *μ*g/ml or 100 *μ*g/ml *Quadrella incana* leaf extract for 3 days. Expression of each protein was normalized to that of GAPDH. The relative band intensity of each protein is shown in the graph below. For each protein, the intensity of the DMSO-treated sample was arbitrarily set as 1. (c) Relative mRNA expression levels of Fubp1 and FoxO1 in proliferating NSPCs treated with DMSO, 20 *μ*g/ml or 100 *μ*g/ml *Quadrella incana* leaf extract for 3 days. Values are means ± s.e.m. (d) Western blot analysis of Prdx3 in proliferating NSPCs after treatment with DMSO or 100 *μ*g/ml *Quadrella incana* leaf extract for 3 days. The relative band intensity of Prdx3 protein is shown in the graph below. The intensity of DMSO-treated sample was arbitrarily set as 1. (e) mRNA expression analysis of SOD2 in proliferating NSPCs treated with DMSO or 100 *μ*g/ml *Quadrella incana* leaf extract for 3 days. Values are means ± s.e.m. (f) Relative ratio of reduced to oxidized glutathione (GSH/GSSG) in NSPCs treated with DMSO or 100 *μ*g/ml *Quadrella incana* leaf extract.

Based on these evidences, we tried to determine protein levels of FoxO1 and FoxO3 in NSPCs treated with *Quadrella incana* leaf extract. Interestingly, *Quadrella incana* extract clearly upregulated FoxO1 in NSPCs. However, FoxO3 abundance was not altered by the extract treatment (Figure 4(a)). Although FoxO1 expression was not induced by a lower concentration (20 *μ*g/ml) of the extract, treatment with 100 *μ*g/ml did increase the levels of FoxO1 protein (Figure 4(b)) and mRNA (Figure 4(c)). FoxO1 immunostaining also showed that FoxO1 was accumulated in *Quadrella incana* leaf extract-treated NSPCs (Supplementary Fig 2A). Because FoxO1 was reported to maintain stemness by upregulating the Notch pathway in adult NSPCs [22], we tested whether FoxO1 induction by the extract also positively controls the Notch pathway. However, *Quadrella incana* leaf extract failed to increase the Csl-mediated Notch reporter activity (Supplementary Fig 2B). In addition, the expression level of Hes1, one of the main target genes of the Notch pathway, was unchanged by treatment with the extract (Supplementary Fig 2C), suggesting that FoxO1 accumulation by *Quadrella incana* leaf extract showed a negligible effect on the Notch pathway.

We next tested whether *Quadrella incana* leaf extract indeed elevated the ROS scavenging capacity of NSPCs through FoxO1 induction. Among FoxO1 target genes, Prdx3 is known to have a prominent role in maintaining the cellular redox balance and mitochondrial functions [24, 25]. In addition, Sod2, another transcriptional target of FoxO1 [24], was reported to scavenge oxygen radicals in NSPCs, leading to cell survival [12]. As expected, FoxO1 accumulation by *Quadrella incana* leaf extract resulted in the upregulation of Prdx3 and Sod2, suggesting that the extract possibly enhances the redox potential of NSPCs (Figures 4(d) and 4(e)). In line with these results, the relative ratio of reduced to oxidized glutathione (GSH/GSSG) was increased by *Quadrella incana* leaf extract, suggesting that the extract possibly upregulated the reducing capacity of NSPCs (Figure 4(f)). Consistently, the extract dramatically suppressed the accumulation of reactive oxygen species inside NSPCs in a dose-dependent manner (Supplementary Fig 3). Together, our results demonstrate that *Quadrella incana* leaf extract could suppress oxidative stress through FoxO1 accumulation, contributing to the redox homeostasis of NSPCs.

### 3.5. *Quadrella incana* Leaf Extract Activated Lactate-AKT-mTOR Axis by Upregulating Glycolytic Flux

**Figure 5:**
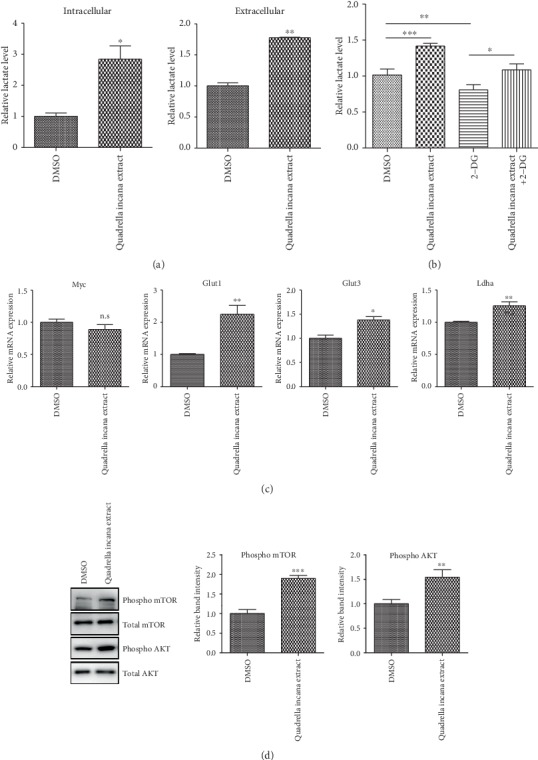
*Quadrella incana* leaf extract upregulated glycolytic pathway, leading to the activation of lactate-AKT-mTOR axis. (a) Relative intracellular or extracellular lactate levels in proliferating NSPCs after treatment with DMSO or 100 *μ*g/ml *Quadrella incana* leaf extract. The relative lactate levels of DMSO-treated cells were set to 1. (b) Relative extracellular lactate levels in proliferating NSPCs in the presence or absence of 2-DG after treatment with DMSO or 100 *μ*g/ml *Quadrella incana* leaf extract. The relative lactate levels of DMSO-treated cells were set to 1. (c) mRNA levels of Myc and glycolytic genes (Glut1, Glut3, and LHDA) in proliferating NSPCs treated with DMSO or 100 *μ*g/ml *Quadrella incana* leaf extract were measured by qRT-PCR. Values are means ± s.e.m. The mRNA levels of DMSO-treated cells were set to 1. (d) Western blot analysis of phospho mTOR and phospho AKT in proliferating NSPCs after treatment with DMSO or 100 *μ*g/ml *Quadrella incana* leaf extract for 3 days. Levels of phospho mTOR and phospho AKT were normalized to total mTOR and total AKT, respectively. The relative band intensities of phospho mTOR and phospho AKT proteins are shown on the right panels. The intensities of DMSO-treated sample were arbitrarily set as 1.

In addition to FoxO1, we also found that Fubp1 was upregulated by *Quadrella incana* leaf extract in a dose-dependent manner (Figures 4(a)-4(c)). A previous study demonstrated that Fubp1 enhances the lactate production, leading to AKT-mTOR activation [26, 27]. Given that oxidative glycolysis is important for the energy metabolism of NSPCs, we first checked the lactate production, the end product of glycolysis, in *Quadrella incana* leaf extract-treated NSPCs. Interestingly, intracellular and extracellular lactate levels were dramatically increased upon *Quadrella incana* leaf extract treatment (Figure 5(a)). Clearly, the extract reversed the lactate level that was inhibited by 2-DG (Figure 5(b)). However, the expression of Myc, a major coordinator of glycolysis, was unchanged by extract treatment, suggesting that other regulators including Fubp1 would be involved in the upregulation of glycolytic activity (Figure 5(c)). Consistent with the upregulated lactate production, the levels of main glycolytic genes such as Glut1, Glut3, and Ldha were significantly upregulated by the extract. When we compared the expression levels of glycolytic genes between undifferentiated NSPCs and differentiated cells, several genes including Glut1, Glut3, Hk1, Hk2, Pkm1, and Ldha appeared to be downregulated upon differentiation (Supplementary Fig 4). Therefore, the upregulation of glycolytic genes by the *Quadrella incana* leaf extract would contribute to the maintenance of undifferentiated status by enhancement of lactate synthesis.

As previously reported, lactate can activate the AKT-mTOR axis [27]. To test whether *Quadrella incana* leaf extract induces the AKT-mTOR axis, phosphorylated levels of AKT and mTOR were determined. As expected, the phosphorylation level of each protein was clearly increased, mirroring that upregulated lactate synthesis presumably contributed to the activation of the AKT-mTOR axis (Figure 5(d)). In line with this result, mitotic marker phospho H3 was upregulated by *Quadrella incana* leaf extract. However, inhibition of PI3K-AKT and mTOR pathway clearly reduced phospho H3 level, confirming that *Quadrella incana* leaf extract contributes to the NSPC growth through AKT-mTOR axis (Supplementary Fig 5). Together, our data collectively suggests that *Quadrella incana* leaf extract enhances the glycolytic flux, leading to the lactate-AKT-mTOR axis activation.

## 4. Discussion

Recent preclinical and clinical studies raised a possibility that grafted NSPCs may replace degenerated cells in neurodegenerative conditions. To use NSPCs in stem cell therapies, the securement of many healthy cells should be a key prerequisite. Given the importance of glycolytic flux and redox balance in the proliferation and stemness of NSPCs, we showed that *Quadrella incana* leaf extract contributed to the maintenance of NSPCs by upregulating glycolysis and redox potential (Supplementary Fig 6).

In this study, we focused on two genes, FoxO1 and Fubp1. While FoxO1 is highly expressed in the proliferating NSPCs, it is downregulated in the early phase of neuronal differentiation [22]. Although FoxO1 was reported to antagonize the differentiation and maintain the homeostasis of adult NSPCs through the cooperation with Notch pathway, our results demonstrated that FoxO1 accumulation upon *Quadrella incana* leaf extract treatment did not enhance the Notch activity. Instead, our study found that the increased FoxO1 upon *Quadrella incana* leaf extract treatment upregulated its target antioxidant genes Prdx3 and Sod2. Therefore, *Quadrella incana* leaf seemed to support the proliferative potential of NSPCs through regulating the cellular redox balance. Previously, we demonstrated that Fubp1 increases the lactate production, leading to the activation of the AKT-mTOR axis, by regulating hexokinase genes. Although we showed that *Quadrella incana* leaf extract promoted the accumulation of Fubp1 in NSPCs and induced the lactate-AKT-mTOR axis, the extract enhanced the expression of Glut1, Glut3, and Ldha, rather than hexokinase genes. Therefore, it would be possible that molecular mechanisms or target gene sets of Fubp1 could be cell-type dependent. Otherwise, it would also be possible that another key glycolysis regulator would be involved in the *Quadrella incana* leaf-mediated lactate-AKT-mTOR axis elevation.

Alterations in NSPC homeostasis followed by reduced generation of newborn neurons are associated with several neurological or neurodegenerative diseases, such as Alzheimer's disease, dementia, and depression. Given the therapeutic capacity of NSPCs, a fundamental understanding of how NSPC fate is tightly regulated would be required. In the present study, we suggested that metabolic flux and redox homeostasis would be critical factors for NSPC cell fate decision. Although we found that *Quadrella incana* leaf extract enhances the NSPC proliferation through regulating these critical factors *in vitro*, we could not verify the biological function of the extract in NSPC proliferation *in vivo*, because pharmacokinetic characteristics of this extract were not analyzed yet. If the *in vivo* physiological function of this extract on NSPC homeostasis is verified in the future, it will be possible to use the *Quadrella incana* leaf extract clinically to boost NSPC proliferation followed by efficient neurogenesis.

Although we did not identify a specific molecule responsible for the bioactivity of *Quadrella incana* leaf extract on NSPCs, there may be positive interactions between components in the crude extract of a given plant. Our findings may be useful for obtaining a sufficient quantity of clinically available NSPCs for therapeutic strategies against acute brain injury or long-term degenerative conditions.

## Figures and Tables

**Table 1:** 

Gene name	Sequence (5′ to 3′)
*Tubb3*	TAGACCCCAGCGGCAACTAT GTTCCAGGTTCCAAGTCCACC
*Fubp1*	GGAACAACACCTGATAGGATAGC GCCAGCCTGAACACTTCGTAG
*FoxO1*	TTCAATTCGCCACAATCTGTCC GGGTGATTTTCCGCTCTTGC
*SOD2*	GTGTCTGTGGGAGTCCAAGG AGCGGAATAAGGCCTGTTGT
*Myc*	TGAAGGCTGGATTTCCTTTG TTCTCTTCCTCGTCGCAGAT
*Glut1*	CAGTTCGGCTATAACACTGGTG GCCCCCGACAGAGAAGATG
*Glut3*	ATGGGGACAACGAAGGTGAC GTCTCAGGTGCATTGATGACTC
*LDHA*	TGTCTCCAGCAAAGACTACTGT GACTGTACTTGACAATGTTGGGA
*Hes1*	CCAGCCAGTGTCAACACGA AATGCCGGGAGCTATCTTTCT
*HK1*	TGCCATGCGGCTCTCTGATG CTTGACGGAGGCCGTTGGGTT
*HK2*	CGGTACACTCAATGACATCCGA TTCACCAGGATGAGTCTGACC
*PGI*	CAAGACGCCCCTGGAGAAGA TCCATGTCACCCTGCTGGAA
*PGK*	GGGTCGTGATGAGGGTGGAC CTGGGCCCACACAATCCTTC
*PKM1*	GCTGTTTGAAGAGCTTGTGC TTATAAGAGGCCTCCACGCT
*LDHB*	CATTGCGTCCGTTGCAGATG GGAGGAACAAGCTCCCGTG
*Rpl32*	AACCCAGAGGCATTGACAAC CACCTCCAGCTCCTTGACAT

## Data Availability

The data used to support the findings of this study are included within the article.

## Supplementary Materials

Supplementary Materials Supplementary Figure 1. Quadrella incana leaf extract facilitates NSPCs proliferation. (A) Cell growth ability was analyzed using CCK-8 assay in proliferating NSPCs treated with DMSO, 40 *μ*g/ml or 100 *μ*g/ml Quadrella incana leaf extract for 3days. Statistical significance was determined by one-way ANOVA. (B) Immunofluorescence analysis of phospho histone 3(pH 3) at Ser10 (red) abundance in DMSO- or Quadrella incana leaf extract-treated NSPCs. Nuclear DAPI (4',6-diamidino-2phenylindole) staining is in blue. Scale bars = 100 *μ*m. (C) The quantification of pH 3-positive cell proportions in DMSO- or Quadrella incana leaf extract-treated NSPCs as in (B). Supplementary Figure 2. Upregulated expression of FoxO1 by Quadrella incana leaf extract did not activate Notch pathway. (A) Enhanced expressions of FoxO1 and Nestin were determined. FoxO1 staining is shown in green and neural stem cell marker Nestin is shown in red. Nuclear DAPI (4',6-diamidino-2-phenylindole) staining is shown in blue. (B) CSL induced luciferase reporter activity was measured in NSPCs with vehicle or 100 *μ*g/ml Quadrella incana leaf extract treatment. (C) Quantitative real-time PCR (qRT-PCR) of Hes1 mRNA in NSPCs with vehicle or 100 *μ*g/ml Quadrella incanaleaf extract treatment under proliferating condition (prol) or differentiating condition (diff). Supplementary Figure 3. Quadrella incana leaf extract suppresses accumulation of reactive oxygen species in NSPCs. NSPCs were treated with DMSO or the indicated concentration of Quadrella incana leaf extract. ROS accumulation was assessed by CellROX staining and fluorescence microscopy 24 hrs after treatment. Scale bars = 200 *μ*m. Supplementary Figure 4. Expression changes of main glycolytic genes during NSPC differentiation. Relative expressions of indicated genes in undifferentiated neural stem/progenitor cells (prol) and differentiated neural cells (diff) were measured by qRT-PCR. Supplementary Figure 5. Quadrella incana leaf extract upregulated AKT and mTOR pathway, leading to the proliferation of NSPCs. (A) Western blot analysis of phospho mTOR, phospho AKT, and phospho H3 in proliferating NSPCs. Levels of total proteins were also detected for normalization. 1 *μ*M LY294002 and 20 nM rapamycin was treated to block the PI3K-AKT pathway and mTOR pathway, respectively. (B) The relative band intensities of phosphor proteins are shown. Supplementary Figure 6. A graphical presentation of NSPC homeostatic regulation by Quadrella incana.
