# Supplementary material for: Quadrella incana (Capparaceae) Leaf Extract Enhances Proliferation and Maintenance of Neural Stem/Progenitor Cells through Upregulating Glycolytic Flux and Redox Potential
Source: Oxid Med Cell Longev. 2020 Apr 25;2020:5963037. doi: 10.1155/2020/5963037 (PMC7201589; doi:10.1155/2020/5963037)
Supplement: Supplementary Materials — Supplementary Figure 1. Quadrella incana leaf extract facilitates NSPCs proliferation. (A) Cell growth ability was analyzed using CCK-8 assay in proliferating NSPCs treated with DMSO, 40 μg/ml or 100 μg/ml Quadrella incana leaf extract for 3days. Statistical significance was determined by one-way ANOVA. (B) Immunofluorescence analysis of phospho histone 3(pH 3) at Ser10 (red) abundance in DMSO- or Quadrella incana leaf extract-treated NSPCs. Nuclear DAPI (4',6-diamidino-2phenylindole) staining is in blue. Scale bars = 100 μm. (C) The quantification of pH 3-positive cell proportions in DMSO- or Quadrella incana leaf extract-treated NSPCs as in (B). Supplementary Figure 2. Upregulated expression of FoxO1 by Quadrella incana leaf extract did not activate Notch pathway. (A) Enhanced expressions of FoxO1 and Nestin were determined. FoxO1 staining is shown in green and neural stem cell marker Nestin is shown in red. Nuclear DAPI (4',6-diamidino-2-phenylindole) staining is shown in blue. (B) CSL induced luciferase reporter activity was measured in NSPCs with vehicle or 100 μg/ml Quadrella incana leaf extract treatment. (C) Quantitative real-time PCR (qRT-PCR) of Hes1 mRNA in NSPCs with vehicle or 100 μg/ml Quadrella incanaleaf extract treatment under proliferating condition (prol) or differentiating condition (diff). Supplementary Figure 3. Quadrella incana leaf extract suppresses accumulation of reactive oxygen species in NSPCs. NSPCs were treated with DMSO or the indicated concentration of Quadrella incana leaf extract. ROS accumulation was assessed by CellROX staining and fluorescence microscopy 24 hrs after treatment. Scale bars = 200 μm. Supplementary Figure 4. Expression changes of main glycolytic genes during NSPC differentiation. Relative expressions of indicated genes in undifferentiated neural stem/progenitor cells (prol) and differentiated neural cells (diff) were measured by qRT-PCR. Supplementary Figure 5. Quadrella incana leaf extract upregulated AKT and mTOR [file 5963037.f1.pdf]

Supplementary Figure 1

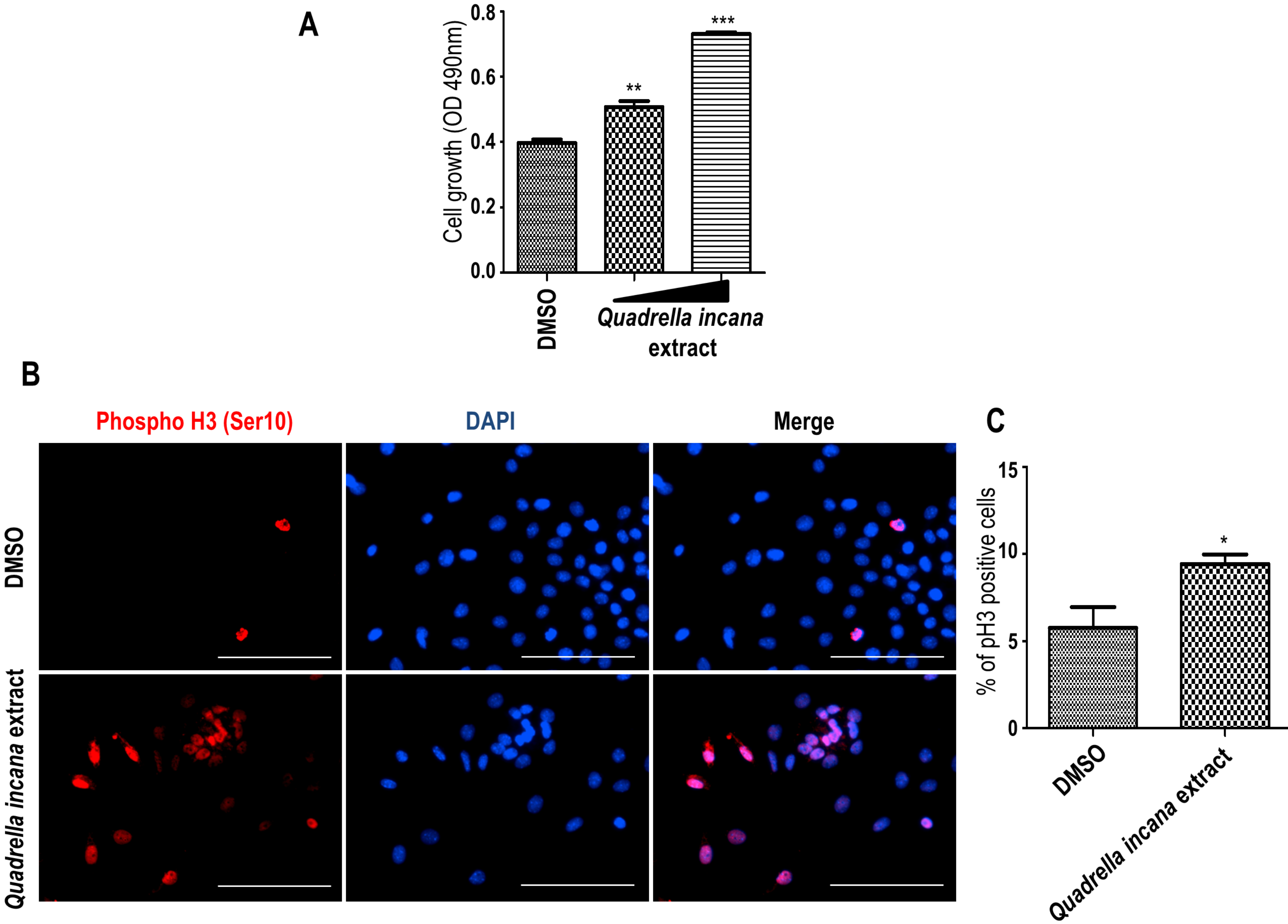

Supplementary Figure 1. *Quadrella incana* leaf extract facilitates NSPCs proliferation. (A) Cell growth ability was analyzed using CCK-8 assay in proliferating NSPCs treated with DMSO, 40μg/ml or 100μg/ml *Quadrella incana* leaf extract for 3 days. Statistical significance was determined by one-way ANOVA, **$P < 0.01$ and ***$P < 0.01$ by post-hoc tests. (B) Immunofluorescence analysis of phosphor histone 3(pH3) at Ser10 (red) abundance in DMSO- or *Quadrella incana* leaf extract-treated NSPCs. Nuclear DAPI (4',6-diamidino-2-phenylindole) staining is in blue. Scale bars = 100 μm. (C) The quantification of pH3-positive cell proportions in DMSO- or *Quadrella incana* leaf extract-treated NSPCs as in (B).

Supplementary Figure 2

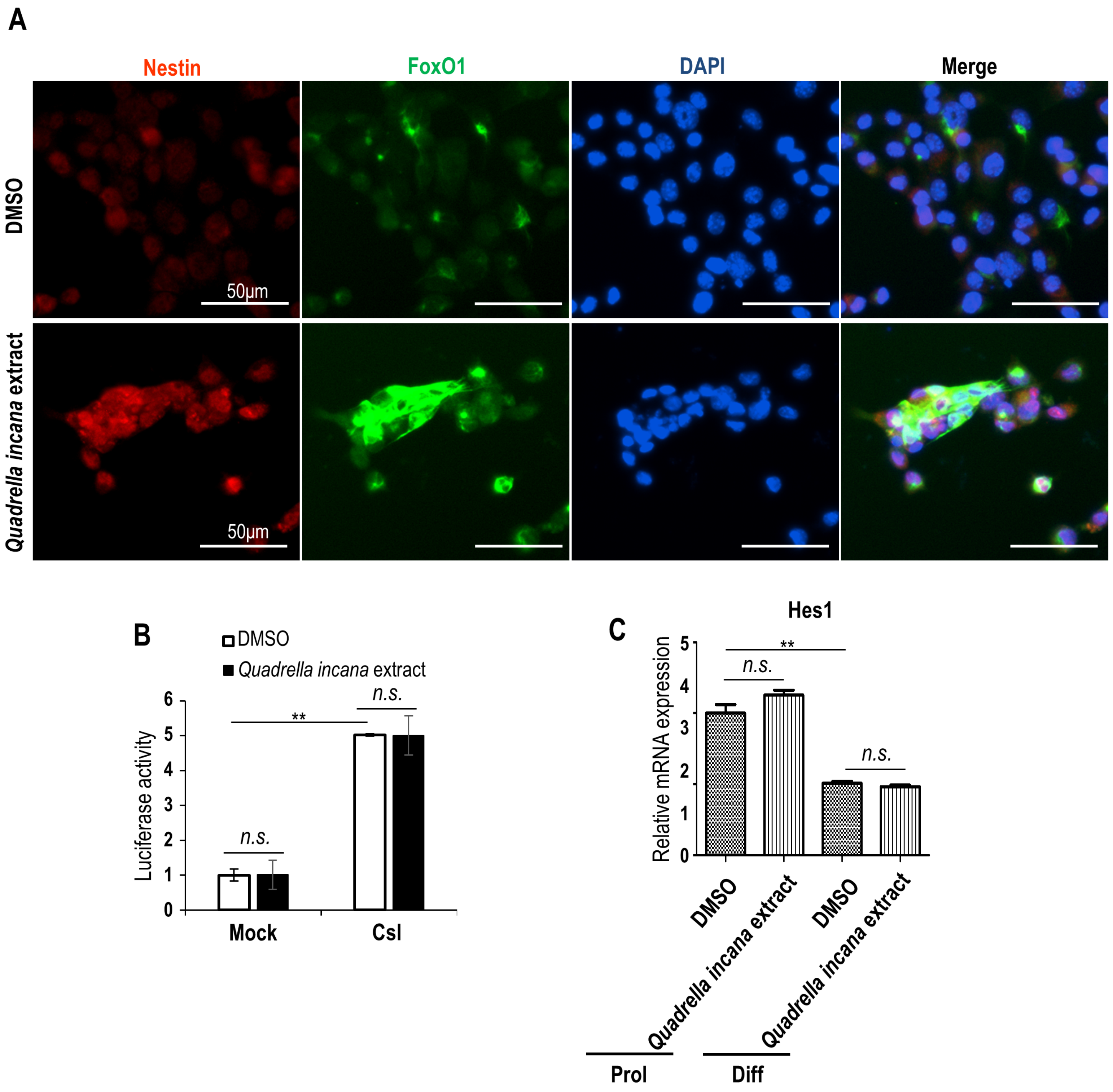

Supplementary Figure 2. Upregulated expression of FoxO1 by *Quadrella incana* leaf extract did not activate Notch pathway. (A) Enhanced expressions of FoxO1 and Nestin were determined. FoxO1 staining is shown in green and neural stem cell marker Nestin is shown in red. Nuclear DAPI (4',6-diamidino-2-phenylindole) staining is shown in blue. (B) CSL induced luciferase reporter activity was measured in NSPCs with vehicle or 100µg/ml *Quadrella incana* leaf extract treatment. (C) Quantitative real-time PCR (qRT-PCR) of Hes1 mRNA in NSPCs with vehicle or 100µg/ml *Quadrella incana* leaf extract treatment under proliferating condition (prol) or differentiating condition (diff).

Supplementary Figure 3

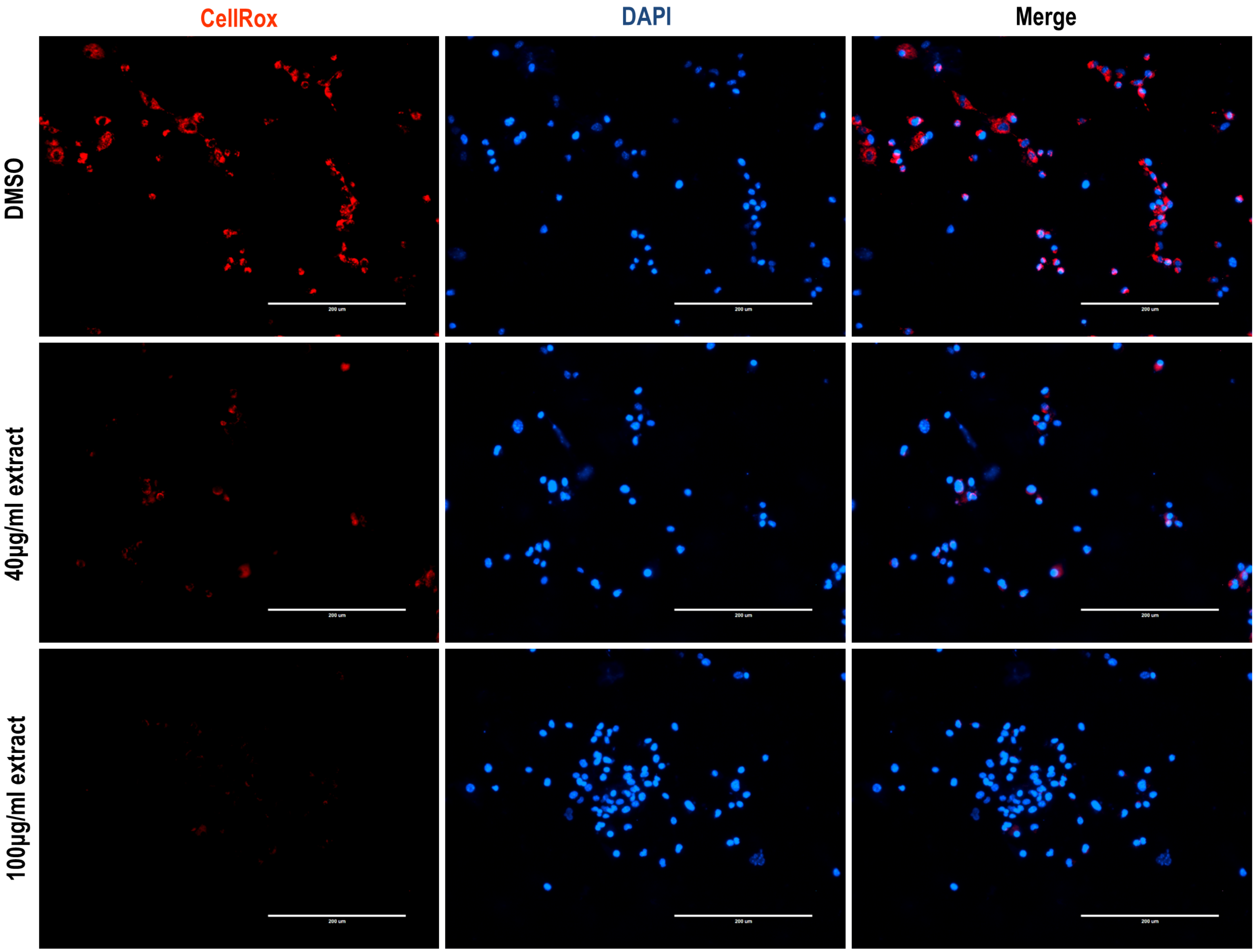

Supplementary Figure 3. ***Quadrella incana*** **leaf extract suppresses accumulation of reactive oxygen species in NSPCs.** NSPCs were treated with DMSO or the indicated concentration of *Quadrella incana* leaf extract. ROS accumulation was assessed by CellROX staining and fluorescence microscopy 24 hrs after treatment. Scale bars = 200 µm.

Supplementary Figure 4

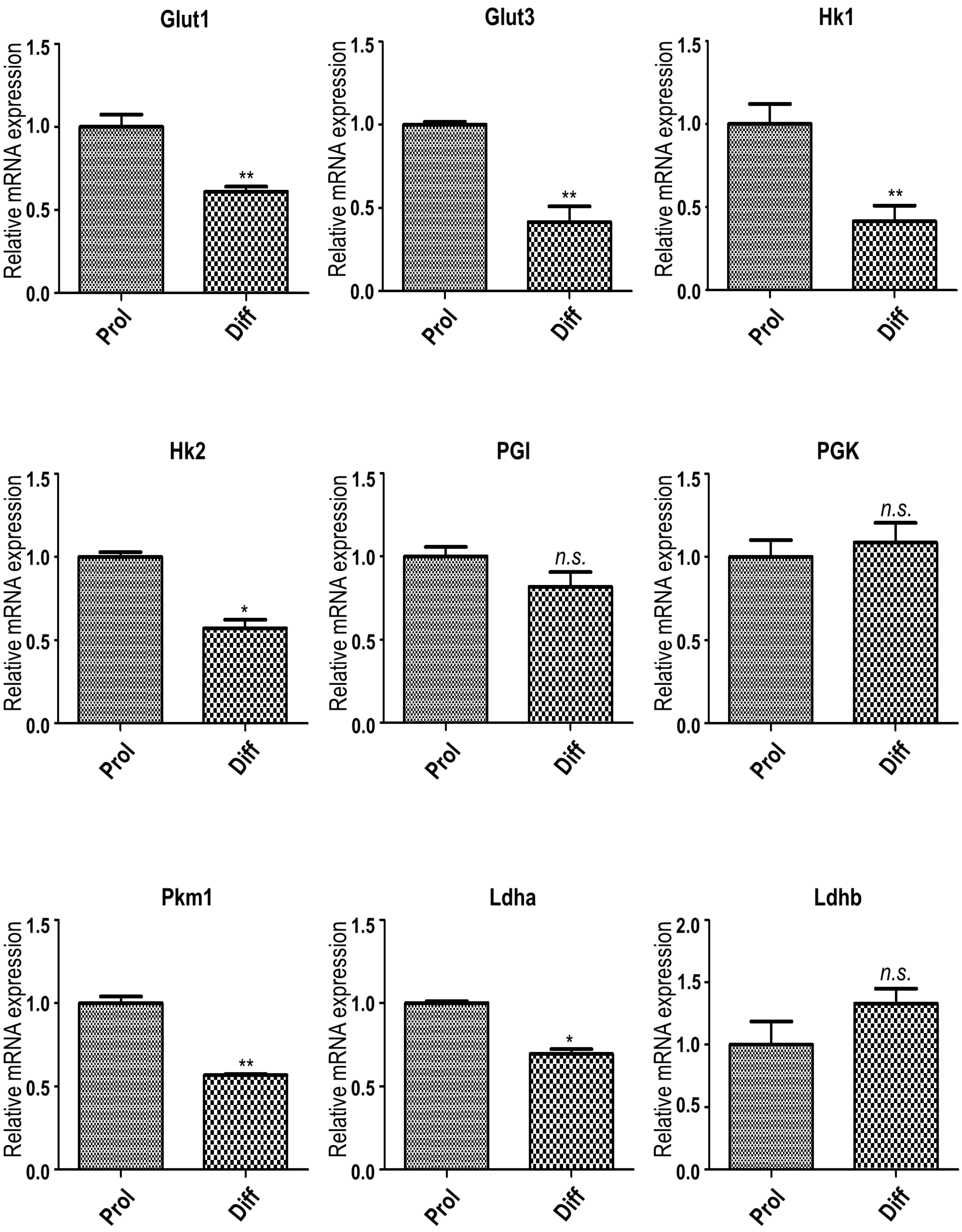

Supplementary Figure 4. Expression changes of main glycolytic genes during NSPC differentiation. Relative expressions of indicated genes in undifferentiated neural stem/progenitor cells (prol) and differentiated neural cells (diff) were measured by qRT-PCR. Values are means ± s.e.m. **P<0.05*, ***P<0.01*.

Supplementary Figure 5

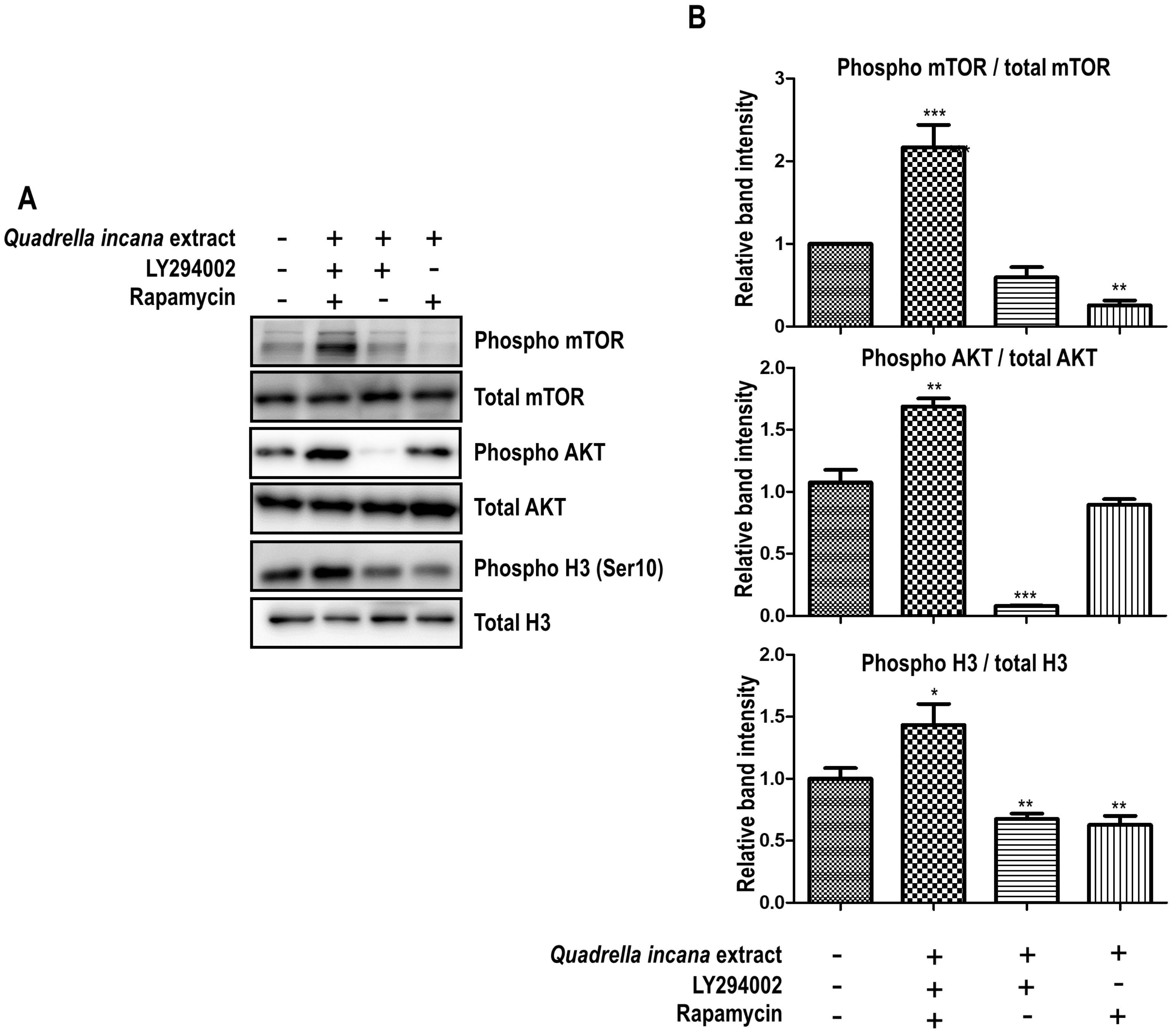

Supplementary Figure 5. ***Quadrella incana*** **leaf extract upregulated AKT and mTOR pathway, leading to the proliferation of NSPCs.** (A) Western blot analysis of phospho mTOR, phospho AKT, and phosphor H3 in proliferating NSPCs. Levels of total proteins were also detected for normalization. 1μM LY294002 and 20nM rapamycin was treated to block the PI3K-AKT pathway and mTOR pathway, respectively. (B) The relative band intensities of phospho proteins are shown. *$*P<0.05$, $**P<0.01$, $***P<0.001$.*

Supplementary Figure 6

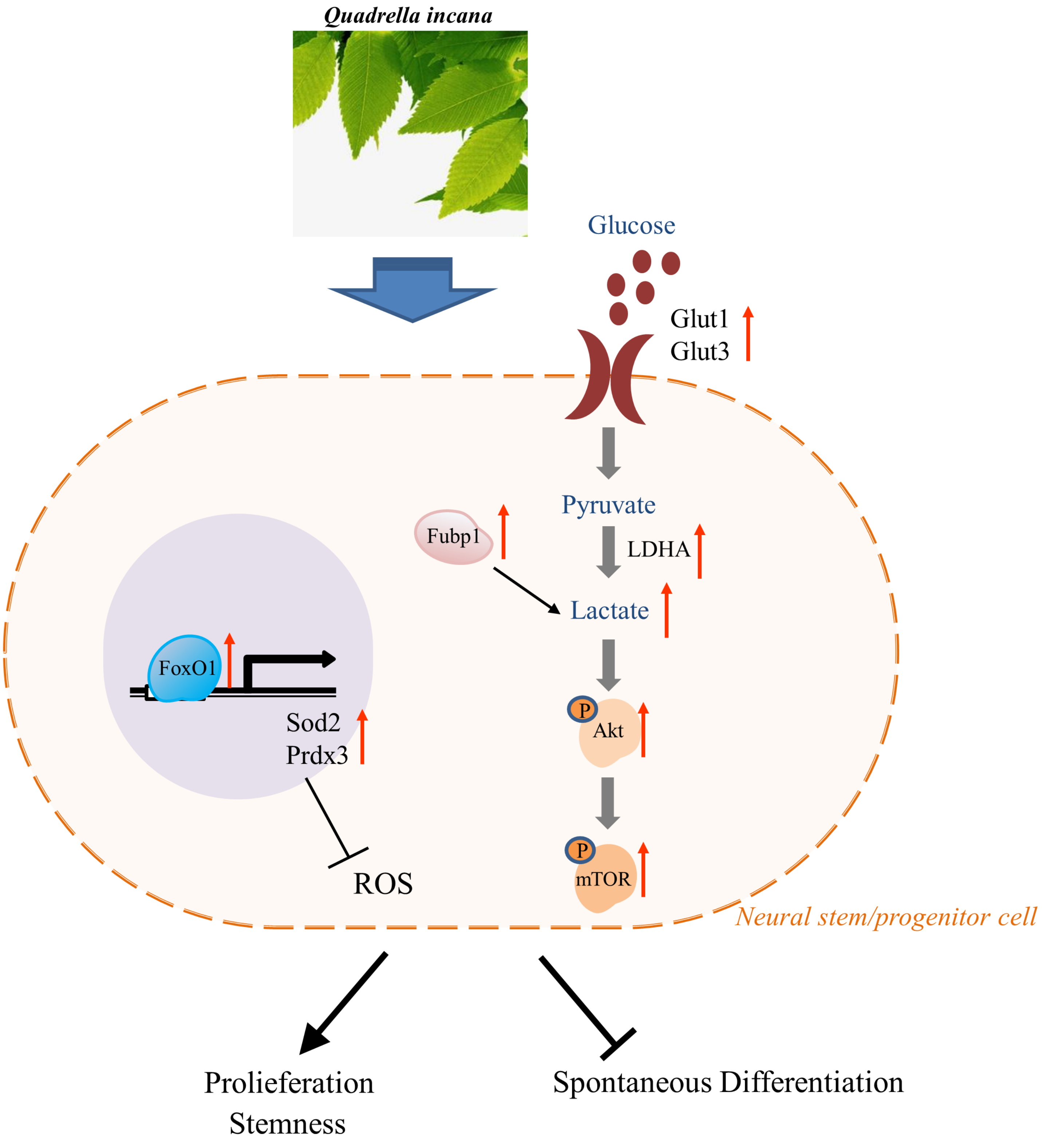

Supplementary Figure 6. A graphical presentation of NSPC homeostatic regulation by *Quadrella incana*
